# Advances in Research of Short Peptides

**DOI:** 10.3390/molecules27082446

**Published:** 2022-04-11

**Authors:** Joanna Bojarska

**Affiliations:** Department of Chemistry, Technical University of Lodz, Zeromskiego 116, 90-924 Lodz, Poland; joanna.bojarska@p.lodz.pl

Short peptides are unique biomolecules, which combine the advantages of classical small molecules and mature proteins and have attracted increasing interest due to their wide range of applications. Recent revolutionary progress in diverse fields of science and biotechnology has helped to overcome the shortcomings of peptides to utilize their full potential. The main aim of the Special Issue on ‘Advances in Research of Short Peptides’ was to build an open platform, where researchers could disseminate cutting-edge findings and directions of studies focused on highlighting the relevance of short peptides in the development of novel therapeutic agents, functional biomaterials, and beyond.

In fact, the purpose has been well achieved. Hard work and interest from leading scientists from diverse research groups from eighteen different countries from all continents of the world (apart from Antarctica, of course) have resulted in the collection of the accepted twenty-five outstanding articles throughout the year. More specifically, this issue comprises fourteen cutting-edge original research papers, eight literature reviews of clear timelines, two short communications, and one brief report, presenting the results of both experimental and theoretical investigations and covering relevant examples of the most recent advances in the research of the short peptides, which are briefly summarized below.

It begins with our global review [1], which is a prelude to the topic of this Issue, as well as the global point of view on frontiers and perspectives of short peptides.

In the time-ongoing pandemic, some contributions are centered on SARS-CoV-2-related studies. First, a nice review written by Matsoukas and co-authors [2] provides a discussion concerning the perspective of angiotensin receptor blockers (ARBs), in terms of short peptides, in COVID-19 therapy.

Odolczyk et al. [3] have designed native (angiotensin-converting enzyme 2) structure-based short peptides, which can be potential protein–protein interaction inhibitors of the SARS-CoV-2 spike protein. Peptides bind to viral protein with a strong affinity.

Besides, Liscano and collaborators [4] present in silico discovery of antimicrobial peptides as an alternative to control SARS-CoV-2. In this course, the authors have screened the antimicrobial peptide database. Generally speaking, amphibian (caerin) peptides show good results. However, new promising potential therapeutic agents need experimental validation.

Another group of papers deals with the significant role of short peptides in the effective fight against bacteria and fungi. Rivera-Sanchez et al. [5] evaluate the antibacterial activity of the antimicrobial peptide, derived from cecropin *D*-like, against multidrug-resistant Gram-negative bacterial strains. A strong effect was observed against *Klebsiella* *pneumonia* and *Pseudomonas* *aeruginosa*.

McMillan and Coombs [6] tell us about examining the natural role of amphibian peptide magainin (derived from the African clawed frog, *Xenopus laevis*). This small antimicrobial (host defense) peptide has potent bioactivity against diverse pathogens. *Batrachochytrium dendrobatidis*, a chytrid fungus, is a good example of the most deleterious amphibious microbe. Nevertheless, populations of the amphibian immune systems, containing magainin, are in decline worldwide. Investigations and understanding of the mechanisms of magainin action would help develop a conservation strategy and effective treatment of infections, and also those caused by other species of amphibians (e.g., the North American green frog, *Rana clamitans*). What is more, studies on the magainin role are crucial to protect also the ecosystems inhabited by amphibian populations.

Yuan and colleagues [7] present a summarized current progress of research on the biosynthesis of pulcherriminic acid by *Bacillus*. Pulcherriminic acid, obtained from cyclic dipeptide cyclo(*L*-Leu-*L*-Leu), is a safe bio-active antibacterial agent with great potential for the industry. The clarification of the regulatory mechanism, structure, and function of the key enzymes concerning the biosynthetic pathways would provide tips for further studies and facilitate the wide applications of this peptide in medicine, food protection, and agriculture.

A major portion of the articles in this collection explore the promising role of short peptides in the treatment of diverse cancers, still a leading cause of deaths all over the world.

Liscano and collaborators [8] focus on the short peptides with dual antimicrobial–anticancer activity. They consider efforts to overcome their limitations, such as poor stability, thanks to nanotechnology. This interesting review is extended to discussion on advances in in silico tools in computational biology useful for the design and study of this kind of peptides, leading to the development of drugs with enhanced bio-activity and improved stability.

Prakash and co-authors [9] evaluate the anti-cancer effect of natural ultra-short peptide carnosine (*β*-alanyl-*L*-histidine). This research group investigates the influence of carnosine on breast, ovarian, colon, and leukemic cancer cell proliferation. Interestingly, significant inhibition is observed in all cases.

Dyniewicz et al. [10] reveal the strong antinociceptive and cytotoxic activity of opioid short peptides modified by hydrazone and hydrazide. The authors observed that the anti-melanoma effect of peptides is correlated with their lipophilicity. 

Hawryłkiewicz and Ptaszyńska [11] discuss the function of gemcitabine peptide-based conjugates in targeted tumor therapy. Notably, gemcitabine is used in a variety of cancers, mainly pancreatic cancer, but has a poor therapeutic index. Conjugations, that can improve parameters, were provided using both cell-penetrating peptides and receptor binding peptides. The authors suggest that the clinical studies are only a matter of time due to numerous benefits of these peptide modifications, such as high stability and specificity, as well as no toxicity.

Mieczkowski et al. [12] synthesized a series of new rigid diketopiperazine-based analogs of tadalafil, determined their crystal structures, and investigated their bioactivity profile. The studies revealed inter alia that bromine-substituted peptides show an enhanced cytotoxic effect in breast cancer cell lines.

Ellert and co-workers [13] provide an intriguing article in which they discuss the evaluation of the activity of NFAT (nuclear factor of activated T cells) proteins in glioma cancer cells, as well as the impact of inhibition of calcineurin/NFAT interaction via the VIVIT peptide. It should be mentioned that the activation of NFAT transcription factors through calcium-dependent phosphatase calcineurin is essential in controlling T cell activation and during carcinogenesis. The authors observed that overexpression of peptide as a fusion protein with a green fluorescent protein decreases the NFAT-based activity and leads to inhibition of the transcription of endogenous NFAT-target genes. In addition, the efficacy of cell-penetrating peptides facilitating peptide delivery to cells across the membrane is analysed. The studies are important mainly from the point of view of the development of short peptides disrupting protein–protein binding at functional sites as an excellent approach to the modulation of protein functions by monitored interference with protein–protein interactions.

A series of papers in this Issue report the role of short peptides in other diseases too.

Kim et al. [14] examine the effect of the wound healing peptide with a sequence of REGRT on atopic dermatitis. The current drugs used in this chronic and common inflammatory skin disease have numerous serious side effects. The findings reveal that peptide reduces the dermatitis score, ear thickness, as well as the level of blood serum IgE and thymic stromal lymphopoietin. The authors suggest that this short peptide could be a new potential drug that overcomes these problems.

Witkiewicz-Kucharczyk et al. [15] use the synthetic model peptide characterizing the tetrathiolate zinc finger motif of the DNA repair protein XPA, to study the reactions of its zinc(II) complex with hydrogen peroxide, as an oxidative agent, and *S*-nitrosoglutathione, as nitrosative stress agent. Moreover, the authors use the Cd(II) substituted complex of XPA with this model peptide to evaluate cadmium assault-related oxidative stress. Notably, XPA is related to *Xeroderma pigmentosa* type A, a genetic disease characterized by a decreased ability to repair DNA damage due to UV hypersensitivity, while tetrathiolate zinc fingers can be targets of oxidative assault under cellular stress conditions.

Wang et al. [16] develop an ultrathin nanofibrous membrane that can either mimic the native fibrous structure of human Bruch’s membrane (BM) or stimulate the survival of retinal pigment epithelial (RPE) cells after functionalization of surface related to fibrous membranes. Results reveal that coated membranes have still the original morphology of nanofibers. The authors conclude that this new biomimetic BM-IBP (integrin-binding peptides)-RPE nanofibrous graft can be a practicable strategy for the success rate of RPE cell transplantation in the treatment of age-related macular degeneration and retinitis pigmentosa.

In a review article deposited by Zimecki and Kaczmarek [17], effects of structural modifications on the immunosuppressive properties of cyclic nonapeptide cyclolinopeptide A and its analogues are considered. These modifications include the incorporation of proteinogenic and non-proteinogenic amino acids, changes in peptide bonds, and so on. Peptides are compared mainly to cyclosporine A. The authors conclude that cyclo-peptides are more potent and less toxic than linear analogues. Interestingly, the Pro-Pro-Phe-Phe sequence is a key for bioactivity. Moreover, derivatives of cyclolinopeptide A have better solubility and lower toxicity than cyclosporine A. They have relevance in the amelioration of autoimmune and inflammatory diseases.

Sobocińska et al. [18] report that synthesized dimers of natural enkephalinase inhibitors, such as opiorphin, sialorphin, and spinorphin) have an anti-inflammatory effect. Nowadays, the treatment of an inflammatory bowel diseases is still one of the biggest challenges in gastroenterology.

The Special Issue further includes interesting papers describing promising peptide-based molecules, such as peptide aptamers and nucleopeptides.

In detail, New and co-workers in their brief report [19] describe the binding interactions of peptide aptamers. Aptamers are synthetic proteins that can specifically bind to target molecules, like antibodies. In this work, the authors confirm, for the first time, the popular hypothesis that linear peptides participate in stronger binding with cyclo-peptides than with linear analogues. Peptide-based aptamers have great potential in either the treatment or diagnosis [1].

Boback et al. [20] describe, in short communication, the design and nature of short guanosine-based nucleopeptides that can form self-assembled structures. Nucleopeptides, even though they have great potential, have been underexplored so far. The authors characterize the structural diversity of these nucleopeptides. The study can be helpful in the further development of applications for nucleopeptides, inter alia as supramolecular catalysts.

Another interesting issue is raised by Caporale and colleagues [21]. The authors describe within their review the lessons learned from studies on peptide–protein interactions in terms of biomedical applications. In other words, they characterize short peptides as ‘tools’ for bio-supramolecular interactions. Notably, the self-recognition and self-assembly of biomolecules are common processes in nature and can allow the building of ordered (nano)structures via non-covalent interactions. Nanostructure-peptide-based (supramolecular) materials offer new tools (nanoparticles, hydrogels, nanofibers, nanotubes) in bio-sciences, due to their attractive features (tuneable bioactivity, target specificity, biocompatibility, high drug-loading capacity, and stimuli-responsive drug delivery at disease sites). The morphology of these bio-materials can be changed inter alia via tuning the type and structure of peptides. Recent progress in studies on peptide-based nanomaterials has visualized their additional potential in advanced applications. A good example can be therapeutic delivery systems, such as drugs loaded to peptide self-assembly nanomaterials, through encapsulation or conjugation. It intensifies the retention effect at the tumor site and in consequence—the uptake of drug rate. Other interesting aspects of peptide-based supramolecular research (e.g., antimicrobial agents, regenerative medicine, tissue engineering, matrices for cell culture), as well as challenges in the development of powerful new therapies using biomaterials, are described in the paper in detail.

Of note, Merski et al. [22] propose a method of identification of short hydrogen-mediated interactions in proteins via ‘heavy atom geometry alone’ (without clear determination of H positions by experimental or theoretical methods). A set of angles can be easily calculated by incorporating unique partner atoms (the atom type of the donor and acceptor heavy atoms). Identification of conserved geometries for H-mediated interactions is possible by comparison of the distance between the donor and the acceptor and these angles to the statistical preferences in the PDB. This approach, utilizing a large amount of available protein crystallographic data, may be helpful in protein design.

Minkiewicz et al. [23] provide the annotation of phosphorylated amino acids and peptides by biochemical codes. Phosphorylation is useful for the improvement in the functional features of food proteins. The latter can be a source of phosphopeptides important for body functions. The authors present simple, standardized, human- and machine-readable codes describing the location of phosphate residues in side chains and the diversity of phosphate residues. The proposed codes can be converted into SMILES representations, and may be used in databases and computational programs that annotate phosphopeptide sequences. Codes can also be useful to describe other types of modifications.

In the frame of short peptide synthesis, two articles have been deposited.

A communication, concerning the solid-phase synthesis of an imidazoline peptidomimetic of an insect pyrokinin neuropeptide, has been submitted by Kaczmarek and colleagues [24]. The authors offer a useful tool to chemists to develop analogues of bioactive peptides with potential selective and/or antagonist properties.

Finally, Ueda et al. [25] in the original research report the successful design and synthesis of helical *N*-terminal *L*-prolyl oligopeptides containing hydrocarbon stapling. Side-chain stapling is an appealing method for the development of stable bio-active peptides. Thus, stapled peptides can be applicable to organocatalysts. The studies indicate the benefits of side-chain stapling related to enhancing catalytic activity.

In summary, the regained and growing interest in short peptides is observed, which is reflected in this Special Issue. As can be seen from the contents of this big collection, each contribution is unique and each valuable, which clearly illustrates the emerging trends in the field of short peptides that will influence the world soon. We are witnessing an evolving revolution in bio-medicine.

To date, numerous new reports have described the great potential of short peptides. Therefore, based on the excellent contributions to this issue, a continuation of the Special Issue related to short peptides and promotion of new research, bringing an update on the advances in research of these fascinating small biomolecules in the future, would be advisable.

I encourage readers to the lecture on all articles deposited in this Issue, which can be accessed at the link: https://www.mdpi.com/journal/molecules/special_issues/Short_Peptides (accessed on 1 April 2022).

I hope that it will stimulate further exciting studies on short peptides, the development of novel concepts and strategies toward a better understanding of bioprocesses leading to effective and safe therapies, including (neuro)regenerative (bio)medicine to improve quality of life, and increase longevity.

## Data Availability

Not applicable.
